# Dietary supplementation with gac (<em>Momordica cochinchinensis</em>) aril powder and oil enhances coloration, hemato-biochemical parameters, and immunity-related gene expression in ornamental goldfish

**DOI:** 10.14202/vetworld.2026.3008-3026

**Published:** 2026-07-17

**Authors:** Anurak Khieokhajonkhet, Wirasinee Noitang, Niran Aeksiri, Narongrit Muangmai, Kumrop Ratanasut, Wilasinee Inyawilert, Kunlayaphat Wuthijaree, Pattaraporn Tatsapong

**Affiliations:** 1 Department of Agricultural Science, Faculty of Agriculture, Natural Resources and Environment, Naresuan University, Phitsanulok 65000, Thailand.; 2 Department of Fishery Biology, Faculty of Fisheries, Kasetsart University, Bangkok 10900, Thailand.

**Keywords:** aquaculture, carotenoids, Carassius auratus, feed additive, gac aril oil, immunity, Momordica cochinchinensis, ornamental fish

## Abstract

**Background and Aim:**

The use of plant-derived functional feed additives has gained increasing attention as a sustainable strategy to improve fish health and reduce dependence on synthetic pigments and chemotherapeutics in aquaculture. Gac (*Momordica cochinchinensis*) arils are rich in carotenoids, flavonoids, and other bioactive compounds with recognized antioxidant and immunomodulatory properties. However, their application in ornamental fish nutrition remains largely unexplored. This study compared the effects of dietary supplementation with gac aril powder (GP) and gac aril oil (GO) on growth performance, pigmentation, hemato-biochemical parameters, liver histology, and immunity-related gene expression in ornamental goldfish (*Carassius auratus*).

**Materials and Methods:**

Five isonitrogenous and isolipidic diets were prepared: a control diet without supplementation, GP at 10 and 30 g/kg (GP10 and GP30), and GO at 5 and 10 g/kg (GO5 and GO10). Goldfish (20 fish per tank; three replicates per treatment) were fed the experimental diets to apparent satiation for 10 weeks in a flow-through system. Growth performance, feed utilization, body coloration, tissue carotenoid deposition, hematological and serum biochemical indices, liver histology, and hepatic expression of tumor necrosis factor-alpha (*TNF-α*), interleukin (*IL-1β*), *IL-10*, *lysozyme*, and heat shock protein 70 (*HSP-70*) were evaluated.

**Results:**

Neither GP nor GO significantly affected growth performance, feed conversion ratio, protein efficiency ratio, somatic indices, whole-body composition, or liver histology. Nevertheless, dietary supplementation significantly improved protein productive value, with the highest value observed in GP30. GO10 markedly enhanced skin redness, particularly in the abdominal and caudal regions, and significantly increased carotenoid accumulation in muscle, skin, liver, and serum, whereas GP30 produced the highest carotenoid deposition in fins. Both GP and GO increased red blood cell count and hemoglobin concentration, while GO supplementation also elevated white blood cell count. GO10 significantly reduced aspartate aminotransferase, total cholesterol, and triglyceride concentrations while increasing high-density lipoprotein cholesterol. Furthermore, GP and GO upregulated *IL-1β*, *IL-10*, and *lysozyme* expression, with GO10 producing the strongest immunostimulatory response, including approximately 20-, 26-, and 36-fold increases in *TNF-α*, *IL-10*, and *lysozyme*, respectively, whereas *HSP-70* expression remained unchanged.

**Conclusion:**

Dietary supplementation with gac aril products, particularly 10 g/kg GO, effectively enhanced pigmentation, carotenoid deposition, hematological and lipid metabolic profiles, and innate immune-related gene expression without compromising growth performance or liver integrity. GO represents a promising natural, multifunctional feed additive that improves the health, coloration, and commercial value of ornamental fish and offers a sustainable alternative to synthetic carotenoids in aquaculture.

## INTRODUCTION

The ornamental fish industry is one of the largest sectors of the global aquaculture trade, with an estimated annual market value of US$15–30 billion [1]. However, intensive commercial production poses significant challenges to maintaining fish welfare under increasingly demanding production systems. Ornamental fish are exposed to multiple stressors, including transportation, handling, high stocking density, increased feeding intensity, and intensive management practices compared with extensive production systems [2, 3]. These stressors induce physiological disturbances, impair growth, reduce skin pigmentation, and increase susceptibility to bacterial, viral, and parasitic infections, ultimately resulting in disease outbreaks and substantial economic losses in commercial aquaculture. Moreover, compromised health and chronic stress adversely affect overall fish welfare. Global restrictions on the use of antibiotics and synthetic chemicals for growth promotion and disease prevention have emerged due to concerns about antimicrobial resistance and their adverse effects on aquatic ecosystems and animal health [4]. Consequently, there is an urgent need to develop sustainable nutritional strategies that improve fish health, disease resistance, and production performance while reducing dependence on synthetic compounds.

Natural immunostimulants have emerged as promising alternatives for enhancing fish health and preventing infectious diseases in aquaculture. These immunostimulants include prebiotics, probiotics, and medicinal plants [5]. Herbal feed additives are widely available and can be incorporated into aquafeeds as whole plants, specific plant parts, or purified extracts. Their natural origin, environmental compatibility, ease of application, and favorable effects on fish welfare make them attractive candidates for sustainable aquaculture [6]. Furthermore, medicinal plants contain diverse bioactive compounds that promote growth, exhibit antimicrobial and antioxidant activities, stimulate immune responses, and improve stress tolerance [5, 6, 9]. Owing to these beneficial biological properties, herbal products have attracted considerable attention as functional feed additives that support sustainable and environmentally responsible aquaculture practices.

The gac fruit (*Momordica cochinchinensis*) is a native Vietnamese fruit belonging to the family Cucurbitaceae [7]. The fruit contains bright red, soft, and sticky arils approximately 1–3 mm thick [8]. Gac fruit contains approximately 5% crude protein, with abundant leucine, arginine, and glutamic acid, and 1%–2% crude fat, whereas the arils contain a substantially higher lipid content of approximately 22.3% [9, 10]. In addition, gac arils are exceptionally rich in bioactive compounds, including α-tocopherol, provitamin A, fatty acids (oleic, palmitic, and stearic acids), β-carotene, lycopene, polyphenols, and flavonoids [11–13]. These compounds contribute to the remarkable biological activities of gac arils, including antioxidant, antimicrobial, anticancer, and anti-inflammatory effects [14, 15]. Because of their exceptionally high phytonutrient content throughout the aril, seed, pulp, and peel, together with their recognized medicinal and pharmaceutical properties, gac fruits have been referred to as "super fruit" and "heaven's fruit" [16]. Among these tissues, the red aril oil represents the most nutrient-dense fraction because it contains abundant oil and exceptionally high concentrations of carotenoids, including β-carotene, lycopene, cryptoxanthin, and zeaxanthin [12, 15]. Recent efforts have focused on developing gac aril powder (GP) and gac aril oil (GO) as natural functional food ingredients and medicinal products. Furthermore, the naturally high lipid content of gac arils facilitates the absorption of carotenoids and other fat-soluble nutrients [17–19]. Consequently, GP and GO have considerable potential as sustainable natural alternatives to synthetic carotenoids in ornamental fish diets while simultaneously improving pigmentation and commercial value. Despite extensive investigations in terrestrial animals, studies evaluating GP and GO in aquaculture remain scarce. To date, only one study has demonstrated that dietary gac ethanol extract enhanced pigmentation in false clownfish (*Amphiprion* *ocellaris*) [20].

Goldfish (*Carassius auratus*), members of the family Cyprinidae and order Cypriniformes, are among the most widely cultured freshwater ornamental fish worldwide. Their commercial value is largely determined by body coloration, with bright red and orange pigmentation representing highly desirable market characteristics [21, 22]. Because goldfish cannot synthesize carotenoids *de novo*, dietary supplementation is essential to maintain and enhance skin pigmentation [21, 22]. This physiological limitation provides an opportunity to improve fish quality through nutritional intervention. Previous studies have demonstrated that dietary supplementation with natural carotenoids and bioactive compounds derived from medicinal plants enhances pigmentation and improves the overall health status of goldfish [23, 24]. Conversely, carotenoid-deficient diets result in progressive fading of skin pigmentation. Moreover, because of their manageable size and ease of maintenance *in vitro*, goldfish are widely used as experimental models in biological research [25]. Given their economic importance in the ornamental fish industry, improving growth performance, coloration, and health through sustainable nutritional approaches remains a priority. Natural herbal feed additives therefore represent environmentally friendly alternatives to synthetic pigments and chemical supplements for ornamental aquaculture.

Although *M. cochinchinensis* has been extensively investigated in humans and terrestrial livestock for its exceptionally high levels of carotenoids and bioactive compounds, its application in aquatic species remains largely unexplored. Existing studies have primarily focused on the nutritional composition and biological activities of gac-derived products or evaluated crude extracts in limited fish species, leaving substantial gaps in understanding their efficacy as functional feed additives in ornamental fish. Furthermore, no previous investigation has systematically compared the biological effects of GP and GO, despite their distinct physicochemical characteristics and potentially different carotenoid bioavailability. In addition, comprehensive evaluations integrating growth performance, pigmentation, carotenoid deposition, hematological and serum biochemical responses, liver histology, and immunity-related gene expression within a single experimental framework are currently lacking. Addressing these knowledge gaps is essential for determining whether different forms of gac-derived products can serve as effective and sustainable alternatives to synthetic carotenoids and chemotherapeutic feed additives in ornamental aquaculture.

Therefore, the present study aimed to comprehensively evaluate the effects of dietary supplementation with GP and GO on growth performance, feed utilization, body pigmentation, tissue carotenoid deposition, hematological and serum biochemical parameters, liver histology, and immunity-related gene expression in goldfish (*C. auratus*). In addition, this study directly compared powder and oil formulations of gac arils to determine their relative biological efficacy and potential differences in carotenoid bioavailability. We hypothesized that dietary GP and GO, particularly GO, given its greater lipid-mediated carotenoid availability, would enhance pigmentation, improve physiological and immune responses, and promote overall health without adversely affecting growth performance or liver integrity, thereby supporting their application as sustainable, multifunctional feed additives for ornamental aquaculture.

## MATERIALS AND METHODS

### Ethical approval

This study strictly followed the 3R principles of replacement, reduction, and refinement. All experimental procedures involving goldfish were reviewed and approved by the National Animal Care and Use Committee, Naresuan University, Phitsanulok, Thailand, under approval number NU-AQ660703. The experiment was also conducted in accordance with the guidelines of the Institute of Animals for Scientific Purpose Development under the National Research Council of Thailand’s Ethics of Animal Experimentation (License No. U1/00704/2558).

All fish handling, acclimatization, feeding, anesthesia, blood sampling, tissue collection, and euthanasia-related procedures were performed by trained personnel using methods designed to minimize stress, pain, and unnecessary harm. Fish were acclimatized before the feeding trial, maintained under controlled water-quality conditions, and monitored daily for health, behavior, feeding activity, and survival. Anesthesia was applied before color measurement, blood sampling, and terminal procedures, and fish welfare was prioritized throughout the 10-week experimental period. No treatment-related mortality, abnormal behavior, or external lesions were observed during the study.

### Study period and location

The feeding trial was conducted over a 10-weeks period from May to July 2024 at the Laboratory of Fish Nutrition, Naresuan University, Phitsanulok, Thailand. Healthy goldfish (*C. auratus*) fingerlings were purchased as mixed-sex individuals from a fish village market in Baan Pong District, Ratchaburi Province, Thailand, and transported to the laboratory for acclimatization and experimental rearing.

### Study design and rearing conditions

Healthy goldfish were initially screened based on active swimming behavior. Fish were acclimatized to laboratory conditions for 2 weeks in 500-L plastic tanks at a stocking density of 1.2 fish/L, with continuous water circulation maintained at a flow rate of 1200 L/h under a natural photoperiod of approximately 12 h light and 12 h dark. During acclimatization, fish were hand-fed a commercial diet containing 70 g/kg crude lipid, 400 g/kg crude protein, and 40 g/kg crude fiber three times daily to apparent satiation.

At the beginning of the feeding experiment, goldfish with an average body weight of 9.02 ± 0.01 g/fish were randomly collected, bulk-weighed, and allocated to each treatment at 20 fish per tank, in triplicate, into 15 glass tanks with a capacity of 150 L, each filled with 120 L of dechlorinated water. The experimental tanks were arranged using a completely randomized design to account for possible positional effects. Each tank was supplied with dechlorinated tap water and continuous aeration through an airstone to maintain adequate oxygenation. Approximately one-third of the water in each tank was siphoned daily after routine cleaning to remove accumulated waste and solid residues. Fish were manually fed the assigned experimental diets to apparent satiation three times daily at 08:30, 12:30, and 17:30 throughout the 10-week feeding trial. Water-quality parameters were monitored daily. Water temperature ranged from 26.4°C to 28.5°C, dissolved oxygen remained above 6.97 mg/L, and pH ranged from 7.61 to 7.71.

### Gac fruit aril powder and oil preparation

Fresh ripe gac fruit (*M. cochinchinensis*) was collected from a home garden and farm at Chay Uncle Garden, Surin Province, Thailand. The fruit was washed twice with running tap water and air-dried. Subsequently, the peel and pulp were cut open, and the seeds were removed to collect the red arils. The collected arils were divided into two portions. The first portion was freeze-dried using a Christ Beta 2–8 LDplus freeze dryer (Martin Christ, Osterode am Harz, Germany) at −80°C for 48 h. The dried gac sample was ground into a fine powder, passed through a 250-µm mesh sieve, and stored in aluminum-plastic zip bags at −20°C until use for feed formulation.

The second portion of gac arils was air-dried at 50°C overnight. The dried arils were processed using a cold screw oil press (Euro Best Technology, Pathum Thani, Thailand). Subsequently, the gac aril extract was frozen at −80°C, concentrated using a Rotavapor R-210 rotary evaporator (Büchi Labortechnik AG, Flawil, Switzerland), and lyophilized using a Christ Beta 2–8 LDplus freeze-dryer (Martin Christ, Osterode am Harz, Germany) to obtain a stable product. The extraction yield of gac oil was approximately 28.7%. The obtained GO was stored in amber reagent glass bottles at −20°C until use.

### Experimental diets

Five isonitrogenous (427.5 g/kg crude protein), isolipidic (91.9 g/kg crude lipid), and isocaloric (18.81 MJ/kg) diets were formulated with graded levels of GP and GO to distinguish the effects of ingredient form and bioactive concentration. A control diet was formulated without GP or GO supplementation, whereas the other four diets included GP at 10 and 30 g/kg and GO at 5 and 10 g/kg, designated GP10, GP30, GO5, and GO10, respectively (Table 1). The dietary inclusion levels of gac products were selected based on a previous study in laying hens [26]. To achieve isonitrogenous diets, wheat flour and fish oil levels were adjusted, while GP and GO levels were increased.

All powdered ingredients were mixed for 15 min using a C-B20G-A1 kitchen mixer (CKI Family, Nonthaburi, Thailand), then fish oil and lecithin were added and mixed for 5 min. Before pelletizing, water was added at 350 mL/kg diet, and the mixture was homogenized for 10 min. The diets were then pelletized into approximately 2-mm pellets using a meat mincer (CKI Family). The experimental diets were air-dried in a UL50 hot-air oven (Memmert GmbH + Co. KG, Schwabach, Germany) at 50°C overnight. Subsequently, the diets were placed in aluminum-plastic zip bags to prevent light exposure and stored at −20°C until use.

### Color determination

At the end of the experiment, three fish from each tank (n = 9) were randomly collected to determine coloration in three body regions: the head, near the upper eye region; the abdominal region, at the lateral line on the dorsal section; and the caudal fin, at the dorsal region of the caudal fin rays. Fish were anesthetized before color measurement. Color was measured on the left side of each fish using a MiniScan EZ 4500L spectrophoto-meter (HunterLab, Reston, VA, USA), with one measurement recorded per body region. The instrument was calibrated using standard white and black calibration plates before analysis. The L* value represents lightness (100 = white, 0 = black), a* indicates redness/greenness, and b* indicates yellowness/blueness, according to the guidelines of the International Commission on Illumination [27].

### Growth performance, feed utilization, and survival

At week 10, fish were starved for 24 h and anesthetized with 30 ppm clove oil solution prepared as a 1:9 mixture of clove oil and ethanol. The average body weight per fish was determined by weighing all fish in each tank collectively and dividing the value by the number of surviving fish. Growth performance and feed utilization parameters were calculated as follows: weight gain (g/fish) = final body weight − initial body weight; specific growth rate (SGR) (%/day) = [(ln final body weight − ln initial body weight)/days] × 100; Feed conversion ratio (FCR) = individual feed intake (g)/individual weight gain (g); protein efficiency ratio = wet weight gain (g)/protein intake (g); and protein productive value (PPV) (%) = protein gain (g)/protein intake (g) × 100. Survival (%) was calculated as 100 × [(final number of fish)/(initial number of fish)].

**Table 1:** Feed formulation and proximate composition of experimental diets supplemented with gac aril powder (GP) and gac aril oil (GO) (dry matter basis).

**Items**	**Control**	**GP10**	**GP30**	**GO5**	**GO10**
Feed formulation (g/kg)					
Fish meal	390	390	390	390	390
Soybean meal	290	290	290	290	290
Gluten meal	80	80	80	80	80
Squid mealᵃ	40	40	40	40	40
Rice bran	35	35	35	35	35
Corn meal	36	36	36	36	36
Wheat flour	40	30	10	40	40
Gac powder	0	10	30	0	0
Gac oil extract	0	0	0	5	10
Fish oilᵇ	45	45	45	40	35
Lysine	8	8	8	8	8
Methionine	7	7	7	7	7
Lecithin	3	3	3	3	3
Vitamin C	6	6	6	6	6
Vitamin premixᶜ	10	10	10	10	10
Mineral premixᵈ	10	10	10	10	10
Total	1000	1000	1000	1000	1000
Proximate composition					
Crude protein	429.0	424.1	428.5	425.9	430.3
Crude fat	91.1	91.2	92.3	93.1	92.1
Ash	132.5	137.6	138.4	134.8	133.3
Dry matter	953.2	946.8	944.3	954.2	955.4
Total carbohydrates	300.6	293.9	285.1	300.4	299.7
β-carotene (mg/kg)ᵉ	0	0.98	2.95	1.94	5.83
Lycopene (mg/kg)ᶠ	0	0.65	1.97	4.01	12.03
Total carotenoids (g/kg)	0.28	0.46	0.71	0.48	0.66
Gross energy (MJ/kg)ᵍ	18.89	18.66	18.66	18.89	18.94

Fish meal, soybean meal, rice bran, and corn meal were purchased from Praepan Animal Feed Corporation, Phitsanulok, Thailand.

ᵃ Squid meal (The Pro-Squid High Protein Concentrate Powder, Bangkok, Thailand).

ᵇ Fish oil (The Origin Nature, Norway).

ᶜ Vitamin premix (Sun-Mix, Munkong Interfood Co., Ltd., Nakhon Pathom, Thailand).

ᵈ Mineral premix (Minmin, Munkong Interfood Co., Ltd., Nakhon Pathom, Thailand).

ᵉ˒ᶠ Dietary β-carotene and lycopene concentrations were estimated based on their respective contents in GP and GO and the inclusion levels of these ingredients in the experimental diets.

ᵍ Gross energy was calculated from the combustion values of macronutrients according to the National Research Council (2011): 23.6 kJ/g protein, 39.5 kJ/g lipid, and 17.2 kJ/g carbohydrate.

### Somatic indices

Three fish were randomly collected from each tank and anesthetized with an overdose of clove oil solution. Each fish was individually weighed and measured for total length to determine condition factor using the following formula: (final body weight × body length³) × 100. Fish were dissected, and the weights of the viscera and liver were recorded to calculate viscerosomatic index (VSI) and hepatosomatic index (HIS) using the following formula: (organ weight/final body weight) × 100.

### Chemical and phytochemical compositions

The proximate composition of whole-body samples was analyzed from the initial fish (10 fish; data not shown), fish at termination (two fish from each tank), and the experimental diets following the standard methods of the Association of Official Analytical Chemists [28]. Dry matter content was determined using the UL50 hot-air oven (Memmert, Schwabach, Germany) at 105°C until a constant weight was achieved. Crude protein content was determined by the Kjeldahl method (N × 6.25) with H₂SO₄ digestion using a Vapodest semi-automatic Kjeldahl system (Gerhardt GmbH & Co. KG, Königswinter, Germany; method 984.13). Crude lipid content was analyzed using the petroleum ether extraction method (method 920.85) with a classic Soxhlet apparatus (Gerhardt). Ash content was determined by incineration at 550°C for 8 h.

Carotenoids, including β-carotene, lycopene, lutein, zeaxanthin, and astaxanthin, were extracted based on a previously described method [29] with slight modifications. Approximately 0.1 g of sample was homogenized in a mortar using a solvent mixture of n-hexane, ethanol, and acetone at a ratio of 1.50:0.75:0.75 until complete extraction. The total carotenoids were then mixed with 5 mL of distilled water and centrifuged at 1,500 × *g* for 10 min at 25°C. The supernatant was analyzed as described previously [30] using a Waters Carotenoids C30 column (4.6 × 150 mm; Waters Corporation, Milford, MA, USA) with an Agilent 1260 high-performance liquid chromate-graphy system (Agilent Technologies Inc., Santa Clara, CA, USA). A gradient elution program was applied using methanol (A) and MTBE (B) as the mobile phases as follows: 0–8 min, 0% B; 8–14 min, 0%–22% B; 14–24 min, 22% B; 24–29 min, 22%–40% B; and 29–33 min, 40% B. The mobile phase was delivered at a flow rate of 1.0 mL/min, with the column temperature maintained at 30°C. The injection volume was 20 µL. Chromatographic peaks were identified by comparing retention times and spectra with authenticated standards. Detection wavelengths were set at 450 nm for β-carotene, 470 nm for lycopene, 445 nm for lutein, 450 nm for zeaxanthin, and 480 nm for quantification of free astaxanthin and its esterified forms.

Total flavonoid content was determined using a colorimetric method [31]. The absorbance of the reaction mixture was measured at 415 nm. Results were expressed as QE/g DW. All chemical reagents and assay kits were purchased from Sigma-Aldrich (St. Louis, MO, USA).

### Total carotenoid content

Total carotenoid content in tissues and experimental diets was determined following the method of Torrissen and Naevdal [32] with slight modifications. Tissue samples, including fin, muscle, skin, and liver, were collected from two fish per tank (n = 6), with approximately 1 g collected from each tissue. Tissue samples were homogenized in darkness in a glass tube with 5 mL of cold acetone containing 1 g of anhydrous sodium sulfate (Na₂SO₄). The extraction solution was covered with aluminum foil to prevent light exposure and maintain sensitivity, then kept at 4°C overnight. The extraction procedure was repeated with an additional 5 mL of extraction solution, bringing the total volume to 10 mL, until complete pigment exhaustion was achieved. The solution was centrifuged at 3500 × *g* for 5 min at 4°C, and the supernatant was analyzed spectrophotometrically at 450 nm using a UV-1800 spectrophotometer (Shimadzu Corporation, Kyoto, Japan). Acetone was used as the blank reference, and measurements were performed using a 1-cm path-length cuvette. Total carotenoid content (µg/g) was determined using the following equation:

Total carotenoids (µg/g) = A × V × 10⁴ / (2500 × W)

where A is the absorbance at 450 nm, V is the extraction volume (mL), and W is the sample weight (g).

Total carotenoid content in serum was determined as described by Barbosa *et al.* [33]. Serum (50 µL; see the “Blood sampling, hematology, and biochemistry analysis” section) was homogenized with ethanol and hexane at a ratio of 4:1 (v/v) in a glass tube covered with aluminum foil to prevent light exposure and maintain sensitivity. The extraction solution was briefly mixed and centrifuged at 4500 × *g* for 10 min at 4°C. The supernatant was analyzed spectrophotometrically at 450 nm, using ethanol and hexane at a 4:1 (v/v) ratio as the reference.

### Blood sampling, hematology, and biochemical analysis

Blood samples were collected from the caudal vein at the same time of day, between 08:00 and 10:00, to minimize diurnal variation. Samples were pooled (n = 3) and collected using a 26-G needle and 1-mL sterile syringe (Nipro Corporation, Osaka, Japan). Fish were fasted for 24 h before sampling. The fish were anesthetized with a 20 ppm clove oil solution, and blood samples were divided into two portions: one containing ethylenediaminetetraacetic acid (EDTA) as an anticoagulant for hematological analysis, and the other without EDTA for biochemical analysis. The second portion was allowed to clot on ice for 2 h and centrifuged at 2,000 × *g* for 15 min at 4°C. Serum from the upper layer was collected and transferred into a fresh tube for serum biochemical analysis.

Hematological parameters, including red blood cell (RBC) count (×10⁶ cells/µL) and white blood cell (WBC) count (×10⁴ cells/µL), were determined using a Neubauer hemocytometer following Rawling *et al.* [34]. Hematocrit (Hct, %) was determined using the microhematocrit method with a DM1424 hematocrit centrifuge (DLAB Scientific Co., Ltd., Beijing, China). Hemoglobin (Hb, g/dL) was analyzed using the colorimetric method with Drabkin’s assay kit (Sigma-Aldrich), and absorbance was measured at 540 nm.

Serum protein content was determined using a commercial colorimetric kit (Sigma-Aldrich). Serum albumin content was determined using the bromocresol green binding method [35]. Serum globulin, alanine transaminase (ALT), aspartate transaminase (AST), alkaline phosphatase (ALP), total cholesterol, triglycerides, high-density lipoprotein cholesterol (HDL-c), and low-density lipoprotein cholesterol (LDL-c) were determined using commercial assay kits (Sichuan Maker Biotechnology Co., Ltd., Chengdu, China) [36] and quantified using a Cobas C311 automated analyzer (Roche Diagnostics, Rotkreuz, Switzerland). Globulin content was calculated using the following formula: total protein content − albumin content.

### Expression of immunity-related genes

**Total RNA extraction and complementary DNA (cDNA) synthesis:** Approximately 2 g of liver tissue was collected for liver morphology analysis, whereas another portion of liver tissue was preserved in RNAlater (Amnion Life Sciences, Cambridgeshire, UK) at −20°C for subsequent total RNA extraction. Total RNA was extracted from liver tissue ground in liquid nitrogen using a pestle and mortar, and the resulting fine powder was dissolved in 1 mL of QIAzol Lysis Reagent (Qiagen, Hilden, Germany). Samples were purified using the RNeasy Mini Kit (Qiagen) according to the manufacturer’s instructions. To avoid genomic DNA contamination, total RNA was incubated at 37°C for 15 min with DNase I (Thermo Fisher Scientific, Waltham, MA, USA). Total RNA quality was quantified using the 260:280 nm ratio with a Synergy H1 Multi-Mode Reader (BioTek Instruments Inc., Winooski, VT, USA). In addition, total RNA quality was assessed using 2% agarose gel electrophoresis. Total RNA was reverse-transcribed into single-strand cDNA using the RevertAid First-Strand cDNA Synthesis Kit (Thermo Fisher Scientific) according to the manufacturer’s protocol.

**Real-time quantitative polymerase chain reaction (qPCR) analysis:** Gene-specific primer sequences for tumor necrosis factor-alpha (*TNF-α*), interleukin-(*IL)-1*β, *IL-10*, lysozyme, and heat shock protein-70 (*HSP-70*) used in this study were adopted from previously validated studies in ornamental goldfish [23, 24] (Table 2). These primer sets were also used to determine amplification efficiencies, which were 98%, 105%, 95%, 91%, and 99%, respectively, whereas the amplification efficiency of the *β-actin* gene was 99%, based on standard calibration curves generated from cycle threshold values of serially diluted samples (R² > 0.91).

qPCR was conducted using the PCRmax ECO48 real-time qPCR system (PCRmax Ltd., Staffordshire, UK) in a final volume of 20 µL containing 1 µL of diluted (100×) cDNA template, 0.4 µL (10 µM) each of forward and reverse primers, 10 µL of Maxima SYBR Green/ROX qPCR Master Mix (Thermo Fisher Scientific), and 8.2 µL of nuclease-free water. The real-time reverse transcription polymerase chain reaction (RT-PCR) program consisted of initial denaturation at 95°C for 10 min, followed by 40 cycles of denaturation at 95°C for 5 s and annealing/extension at 59°C for 40 s. All real-time RT-PCR samples were analyzed in triplicate, resulting in nine replicates in total. Gene-specific primers targeting *TNF-α*, *IL-1β*, *IL-10*, lysozyme, and *HSP-70* in goldfish were used, with expression levels normalized to *β-actin* and quantified using the 2^−ΔΔCt method.

**Table 2:** Primer sequences used in this study.

**Gene**	**Primer**	**Sequence (5′–3′)**	**Reference**
TNF-α	Forward	CATTCCTACGGATGGCATTTACTT	[23]
	Reverse	CCTCAGGAATGTCAGTCTTGCAT	
IL-1β	Forward	GATGCGCTGCTCAGCTTCT	[23]
	Reverse	AGTGGGTGCTACATTAACCATACG	
IL-10	Forward	CAAGGAGCTCCGTTCTGCAT	[23]
	Reverse	TCGAGTAATGGTGCCAAGTCATCA	
Lysozyme	Forward	GTATCTTCAAGCGAGAGGGACT	[24]
	Reverse	CCCTGTGGGTCTTATACTTACTC	
HSP-70	Forward	GGCAGAAGGTGACAAATGCA	[23]
	Reverse	TGGGCTCGTTGATGTTCTCA	
β-actin	Forward	GATGCGGAAACTGGAAAGGG	[23]
	Reverse	ATGAGGGCAGAGTGGTAGACG	

### Liver morphology

Liver tissues from two fish per tank (n = 6) were fixed in cold, approximately 4°C, 10% neutral buffered formalin [10% (v/v) formalin containing 9 g/L NaCl and 12 g/L Na₂HPO₄, adjusted to pH 7.2–7.4]. The liver from each fish was trimmed into three pieces and processed for histological examination. After fixation, tissue samples were dehydrated in a graded ethanol series, embedded in Paraplast medium (Leica Microsystems, Nussloch, Germany), and cut into 5–6-µm sections using a Leica RM2235 rotary microtome (Leica Biosystems, Nussloch, Germany). Tissue sections were stained with hematoxylin for 3 min, rinsed, differentiated, and counterstained with eosin for 1 min before dehydration and mounting. Sections were examined and imaged using an Olympus BX40 light microscope (Olympus Corporation, Tokyo, Japan). Histopathological alterations were semi-quantitatively scored for lesion severity on a scale of 0–3, where 0 = normal, 1 = mild, 2 = moderate, and 3 = severe.

### Statistical analysis

The SPSS statistical software package (SPSS Inc., Chicago, IL, USA) was used for statistical analyses. Before analysis, data normality and homogeneity of variance were assessed using the Shapiro–Wilk and Levene’s tests, respectively. The effects of dietary supplementation with GP and GO on all measured variables were evaluated using a one-way analysis of variance. Significant treatment effects (p < 0.05) were further analyzed using Tukey’s post hoc test to compare means among experimental groups. Results are presented as mean ± SD. The sample size was determined based on previous ornamental fish feeding studies with similar experimental designs and measured parameters [23], while also considering animal welfare and experimental feasibility.

## RESULTS

### Growth performance, feed utilization, and survival

To our knowledge, this is the first report demonstrating that GO outperforms GP in improving carotenoid deposition and immunity-related gene expression in fish. All experimental groups showed survival rates exceeding 96.67% (Supplementary Figure 1C), with no significant differences among dietary treatments (p > 0.05; Table 3). Growth performance, including final body weight, weight gain, and SGR, and feed utilization, including FCR and PER, were not significantly affected by dietary supplementation with GP or GO (Table 3). Nevertheless, the highest weight gain and SGR and the lowest FCR were observed in the GP30 group (p > 0.05; Supplementary Figures 1A and B). Interestingly, dietary supplementation with GP10, GP30, and GO5 significantly affected PPV compared with the control group (p = 0.001). The highest PPV value was recorded in the GP30 group, followed by the GO5 and GP10 groups. No adverse effects, abnormal behavior, external lesions, or treatment-related mortality were observed. Fish in all groups remained healthy and exhibited normal feeding and swimming behavior throughout the experiment.

**Table 3:** Growth performance, feed utilization, and survival of goldfish fed diets supplemented with gac aril powder (GP) and gac aril oil (GO) for 10 weeks.

**Parameters**	**Control**	**GP10**	**GP30**	**GO5**	**GO10**	**p-value**
Initial body weight (g/fish)	9.02 ± 0.00	9.02 ± 0.01	9.03 ± 0.00	9.02 ± 0.01	9.02 ± 0.01	0.315
Final body weight (g/fish)	16.65 ± 0.58	17.89 ± 1.57	18.81 ± 0.82	18.65 ± 0.73	17.65 ± 0.93	0.130
Weight gain (%)	84.17 ± 6.46	98.28 ± 17.47	108.38 ± 8.96	106.77 ± 8.15	95.65 ± 10.32	0.133
Specific growth rate (%/day)	0.88 ± 0.05	0.97 ± 0.13	1.05 ± 0.06	1.04 ± 0.06	0.96 ± 0.08	0.127
Feed conversion ratio	3.42 ± 0.21	3.30 ± 0.62	2.96 ± 0.15	3.15 ± 0.12	3.29 ± 0.26	0.512
Protein efficiency ratio	0.63 ± 0.02	0.73 ± 0.12	0.79 ± 0.04	0.71 ± 0.03	0.69 ± 0.03	0.160
Protein productive value (%)	9.29 ± 0.20ᵇ	14.47 ± 1.23ᵃ	15.01 ± 1.45ᵃ	14.67 ± 2.34ᵃ	8.67 ± 2.29ᵇ	0.001
Survival (%)	96.67 ± 2.89	100.00 ± 0.00	100.00 ± 0.00	98.33 ± 2.83	96.67 ± 2.89	0.233

Data are presented as mean ± SD (20 fish per replicate; n = 3 for growth performance, feed utilization, and survival). Means within the same row with different superscript letters (ᵃ, ᵇ) differ significantly (p < 0.05). SD = Standard deviation.

### Somatic indices and whole-body composition

Dietary supplementation with GP and GO did not significantly affect condition factor, HSI, or VSI in goldfish (p > 0.05; Table 4). Similarly, dietary GP and GO supplementation did not alter dry matter, crude protein, crude fat, or ash content compared with the control group (p > 0.05).

### Body coloration

The coloration of goldfish fed different levels of GP and GO is shown in Table 5. Dietary GP and GO supplementation significantly affected the L* value of the head region, with higher values observed in the GO5 and GO10 groups than in the control group (p = 0.015). However, the L* value was not significantly affected by GP10 or GP30 supplementation (p > 0.05). Similarly, the L* value of the abdominal region was significantly increased in the GO10 group, whereas the L* value of the tail region was significantly increased in the GO5 and GO10 groups. The a* value was significantly increased in the head, abdominal, and tail regions of goldfish. The highest a* values were observed in the GO5, GO10, and GO10 groups for the head, abdominal, and tail regions, respectively (p < 0.05). The b* value of the head region differed significantly in goldfish fed GP10, GP30, and GO10 compared with the control group. Similarly, the GP10 group showed a significantly higher b* value in the abdominal region than the control group, whereas the b* value in the tail region was not affected by dietary GP or GO supplementation (p > 0.05).

**Table 4:** Somatic indices and whole-body composition of goldfish fed diets supplemented with gac aril powder (GP) and gac aril oil (GO) for 10 weeks.

**Items**	**Control**	**GP10**	**GP30**	**GO5**	**GO10**	**p-value**
Somatic indices						
Condition factor (g/cm³)	3.95 ± 0.61	4.00 ± 0.88	4.21 ± 0.53	4.60 ± 1.09	4.24 ± 1.14	0.677
Hepatosomatic index (%)	1.26 ± 0.84	1.94 ± 0.71	1.24 ± 0.56	1.24 ± 0.44	1.43 ± 0.23	0.080
Viscerosomatic index (%)	11.02 ± 2.04	9.76 ± 1.30	9.65 ± 0.84	10.91 ± 1.41	10.94 ± 1.21	0.342
Whole-body composition (%)						
Dry matter	29.43 ± 1.69	35.07 ± 1.68	32.34 ± 1.77	33.71 ± 1.21	34.34 ± 0.46	0.063
Crude protein	12.23 ± 0.91	12.53 ± 0.70	13.39 ± 1.01	13.18 ± 0.13	11.34 ± 0.48	0.106
Crude fat	19.06 ± 0.84	19.18 ± 0.27	19.35 ± 0.57	20.29 ± 0.58	19.26 ± 0.40	0.728
Ash	1.88 ± 0.67	2.36 ± 0.13	2.40 ± 0.20	2.14 ± 0.14	2.22 ± 0.04	0.373

Data are presented as mean ± SD (n = 9 for somatic indices; n = 3 for whole-body composition, with two fish pooled per replicate).

**Table 5:** Color parameters of the head, abdominal region, and tail of goldfish fed diets supplemented with gac aril powder (GP) and gac aril oil (GO) for 10 weeks.

**Parameters**	**Control**	**GP10**	**GP30**	**GO5**	**GO10**	**p-value**
Head						
Luminosity (L*)	32.25 ± 2.07ᵇ	33.44 ± 3.87ᵇ	37.66 ± 2.08ᵃᵇ	39.90 ± 1.70ᵃ	40.12 ± 0.51ᵃ	0.015
Redness (a*)	8.75 ± 2.14ᵇ	17.48 ± 1.52ᵃ	19.51 ± 1.83ᵃ	21.31 ± 2.04ᵃ	18.94 ± 0.50ᵃ	<0.001
Yellowness (b*)	29.44 ± 1.59ᵇ	39.35 ± 2.31ᵃ	35.69 ± 6.42ᵃ	31.93 ± 1.59ᵃᵇ	41.38 ± 3.03ᵃ	0.001
Abdominal region						
Luminosity (L*)	61.73 ± 5.11ᵃᵇ	55.62 ± 3.36ᵇ	55.64 ± 1.57ᵇ	55.38 ± 1.62ᵇ	64.27 ± 1.97ᵃ	<0.001
Redness (a*)	8.08 ± 0.88ᶜ	18.17 ± 0.83ᵃᵇ	18.79 ± 1.29ᵃᵇ	16.93 ± 2.31ᵇ	21.04 ± 0.80ᵃ	<0.001
Yellowness (b*)	35.49 ± 3.93ᵇᶜ	47.37 ± 0.87ᵃ	43.43 ± 2.98ᵃᵇ	41.41 ± 5.62ᵃᵇᶜ	31.90 ± 4.05ᶜ	0.004
Tail						
Luminosity (L*)	37.68 ± 2.70ᵃᵇ	33.91 ± 0.56ᵇ	36.26 ± 2.84ᵃᵇ	43.05 ± 3.92ᵃ	41.07 ± 1.12ᵃ	0.009
Redness (a*)	16.28 ± 1.96ᵇ	23.64 ± 1.07ᵃ	22.34 ± 1.13ᵃᵇ	23.05 ± 1.88ᵃ	26.03 ± 4.27ᵃ	0.006
Yellowness (b*)	32.07 ± 0.97	30.96 ± 0.76	35.77 ± 4.75	33.44 ± 3.49	32.25 ± 2.73	0.390

Data are presented as mean ± SD (n = 9; three fish per tank). Means within the same row with different superscript letters (ᵃ–ᶜ) differ significantly (p < 0.05).

### Total carotenoid content

The total carotenoid content of goldfish fed different levels of GP and GO is shown in Table 6. Total carotenoid contents in various tissues were affected by dietary GP and GO supplementation. Dietary supplementation with GP and GO increased the total carotenoid content in fins, with the highest level observed in fish fed GP30, followed by the GO10 group (p < 0.05). Fish fed GO-supplemented diets showed significantly higher total carotenoid contents in the muscle, liver, and serum than fish fed GP-supplemented diets and the control diet (p < 0.05).

**Table 6:** Total carotenoid content in various tissues of goldfish fed diets supplemented with gac aril powder (GP) and gac aril oil (GO) for 10 weeks.

**Items**	**Control**	**GP10**	**GP30**	**GO5**	**GO10**	**p-value**
Fin (µg/g)	168.70 ± 6.75ᶜ	157.15 ± 5.51ᶜ	206.39 ± 10.66ᵃ	173.25 ± 10.01ᵇᶜ	195.82 ± 14.11ᵃᵇ	0.001
Muscle (µg/g)	53.63 ± 1.49ᶜ	51.97 ± 1.81ᶜ	53.92 ± 0.88ᶜ	61.95 ± 1.27ᵇ	72.88 ± 1.79ᵃ	<0.001
Skin (µg/g)	152.25 ± 14.20ᵇ	152.25 ± 10.96ᵇ	163.45 ± 13.03ᵇ	254.10 ± 30.26ᵃ	280.00 ± 16.07ᵃ	0.026
Liver (µg/g)	20.62 ± 1.99ᵈ	21.49 ± 1.19ᵈ	31.99 ± 2.24ᶜ	37.45 ± 2.26ᵇ	52.43 ± 4.12ᵃ	<0.001
Serum (µg/mL)	1.35 ± 0.63ᵇ	1.55 ± 0.24ᵃᵇ	1.72 ± 0.23ᵃᵇ	1.75 ± 0.24ᵃᵇ	1.95 ± 0.20ᵃ	0.019

Data are presented as mean ± SD (n = 6; two fish per tank).

Means within the same row with different superscript letters (ᵃ–ᵈ) differ significantly (p < 0.05).

### Hematological and biochemical parameters

RBC and Hb levels increased significantly with increasing dietary GP and GO levels. Dietary supplementation with both GP and GO resulted in significantly higher RBC and Hb levels (Table 7). Dietary inclusion of GO also significantly increased WBC levels (p < 0.05); however, Hct did not differ significantly among dietary treatments (p > 0.05). Total protein, albumin, globulin, albumin:globulin ratio, ALT, ALP, and LDL-c levels did not differ significantly in response to dietary supplementation with GP or GO (p > 0.05). AST levels were affected by dietary GP and GO supplementation; specifically, AST levels were markedly lower in GO-supplemented groups than in the GP and control groups (p < 0.05). The results also showed reduced total cholesterol and triglyceride levels, with the lowest values observed in the GO10 group (p < 0.05). In addition, HDL-c levels were significantly increased in fish fed GO5 and GO10 diets (p < 0.05).

**Table 7:** Hematological and biochemical parameters of goldfish fed diets supplemented with gac aril powder (GP) and gac aril oil (GO) for 10 weeks.

**Blood parameters**	**Control**	**GP10**	**GP30**	**GO5**	**GO10**	**p-value**
Hematological parameters						
Red blood cells (×10⁶ cells/µL)	9.99 ± 0.84ᶜ	14.03 ± 0.67ᵇ	14.81 ± 1.48ᵇ	18.25 ± 0.73ᵃ	20.03 ± 1.76ᵃ	<0.001
White blood cells (×10³ cells/µL)	13.73 ± 1.27ᵇ	15.09 ± 2.87ᵃᵇ	20.88 ± 5.22ᵃᵇ	21.85 ± 2.32ᵃ	22.96 ± 1.49ᵃ	0.011
Hematocrit (%)	31.67 ± 2.98	37.22 ± 4.86	37.66 ± 5.75	39.00 ± 6.40	38.55 ± 8.80	0.306
Hemoglobin (g/dL)	12.98 ± 1.37ᶜ	14.61 ± 0.73ᵇᶜ	15.72 ± 1.19ᵃᵇ	17.26 ± 1.06ᵃ	16.15 ± 0.89ᵃᵇ	<0.001
Biochemical parameters						
Total protein (g/dL)	2.97 ± 0.23	2.91 ± 0.00	2.88 ± 0.23	3.16 ± 0.36	3.16 ± 0.37	0.744
Albumin (g/dL)	1.16 ± 0.08	1.17 ± 0.06	1.23 ± 0.06	1.36 ± 0.16	1.28 ± 0.08	0.335
Globulin (g/dL)	1.81 ± 0.16	1.74 ± 0.06	1.64 ± 0.17	1.80 ± 0.19	1.88 ± 0.28	0.784
Albumin:globulin ratio	0.64 ± 0.01	0.66 ± 0.06	0.75 ± 0.04	0.75 ± 0.00	0.68 ± 0.05	0.151
Aspartate transaminase (U/L)	60.61 ± 9.05ᵃ	33.26 ± 4.45ᵇ	33.69 ± 5.40ᵇ	27.55 ± 1.34ᵇ	27.50 ± 3.33ᵇ	0.007
Alanine transaminase (U/L)	16.56 ± 6.45	18.71 ± 4.90	18.66 ± 2.04	13.64 ± 2.12	17.63 ± 0.63	0.687
Alkaline phosphatase (U/L)	29.62 ± 1.95	32.27 ± 2.91	31.17 ± 2.76	23.74 ± 2.36	30.61 ± 2.14	0.092
Cholesterol (mg/dL)	326.11 ± 5.75ᵃ	300.60 ± 6.36ᵇ	310.12 ± 4.21ᵃᵇ	301.07 ± 8.58ᵇ	264.96 ± 4.07ᶜ	0.013
Triglycerides (mg/dL)	198.10 ± 14.01ᵃ	189.67 ± 6.41ᵃ	164.67 ± 23.43ᵃᵇ	174.45 ± 22.58ᵃᵇ	122.33 ± 7.28ᵇ	0.033
High-density lipoprotein cholesterol (mg/dL)	57.04 ± 3.73ᵈ	67.30 ± 5.79ᶜ	75.28 ± 4.43ᵇ	85.24 ± 8.49ᵃ	88.31 ± 1.63ᵃ	0.043
Low-density lipoprotein cholesterol (mg/dL)	204.59 ± 6.48	195.81 ± 0.57	202.18 ± 4.29	206.14 ± 15.54	193.02 ± 7.17	0.552

Data are presented as mean ± SD (n = 3; four fish were pooled per replicate).

Means within the same row with different superscript letters (ᵃ–ᵈ) differ significantly (p < 0.05).

### Immunity-related gene expression

**Figure 1:**
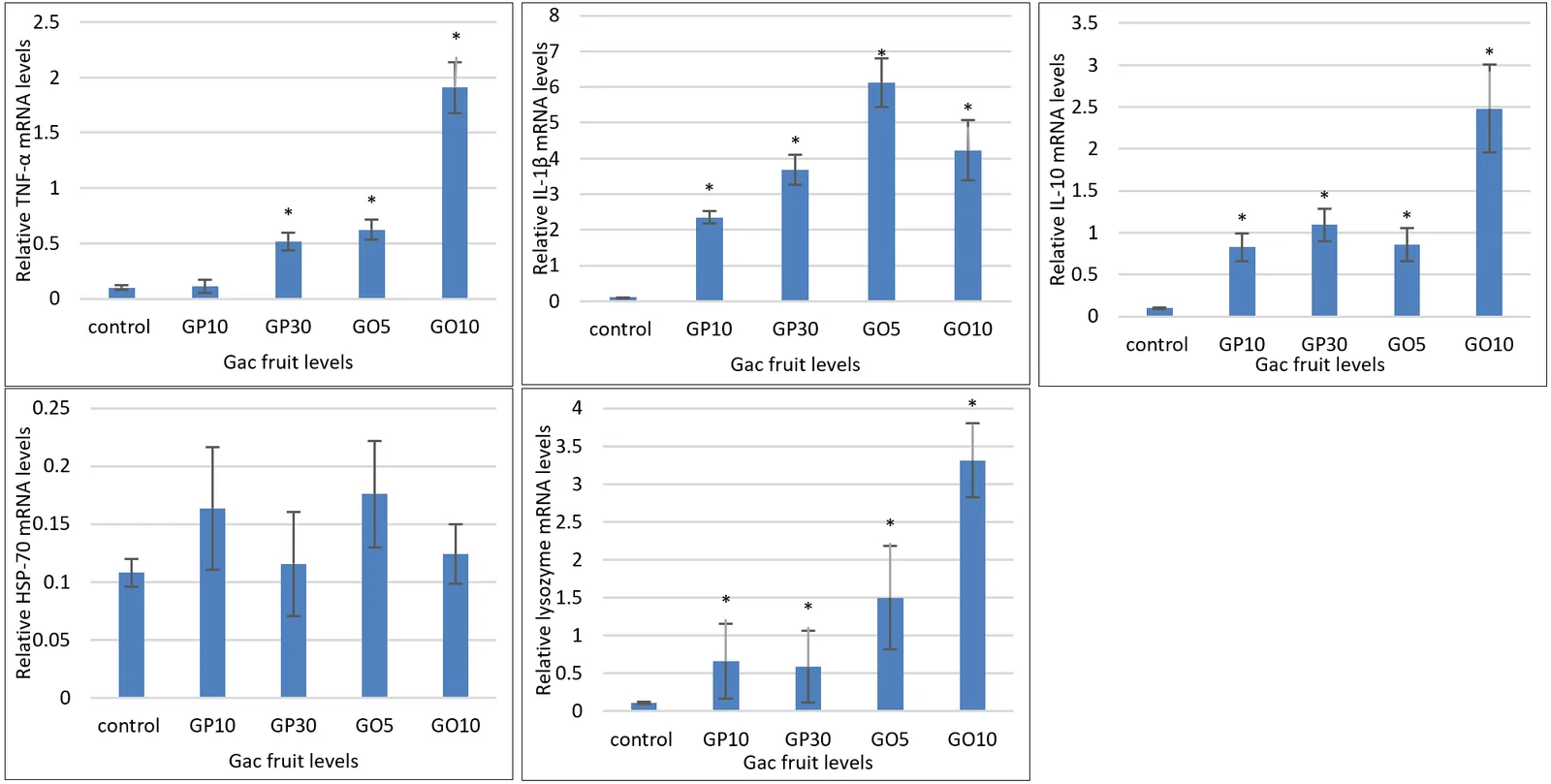
(A) Relative expression of tumor necrosis factor-alpha. (B) Relative expression of interleukin-1β. (C) Relative expression of interleukin-10. (D) Relative expression of heat shock protein-70. (E) Relative expression of lysozyme in the liver of goldfish fed diets supplemented with gac aril powder and gac aril oil. Data are presented as mean ± SD (n = 3), representing three biological replicates per treatment. Different lowercase letters indicate significant differences among treatments (p < 0.05).

Compared with the control group, all GP- and GO-supplemented groups exhibited significantly higher expression levels of *IL-1*β, *IL-10*, and lysozyme (p < 0.05; Figures 1B, C, and E). The highest expression levels of *IL-10* and lysozyme transcripts were observed in the GO10 group, showing a 24.5-fold increase compared with the control group. The GO5 and GO10 groups showed higher *IL-1*β expression than all other groups (p < 0.05). However, *HSP-70* expression was not altered by dietary supplementation with GP or GO (p > 0.05; Figure 1D). The expression of *TNF-α* mRNA was upregulated in the GP30, GO5, and GO10 groups (p < 0.05), with 6-, 7-, and 18-fold increases, respectively, compared with the control group; however, no change was observed in the GP10 group (p > 0.05; Figure 1A).

### Liver histology

**Figure 2:**
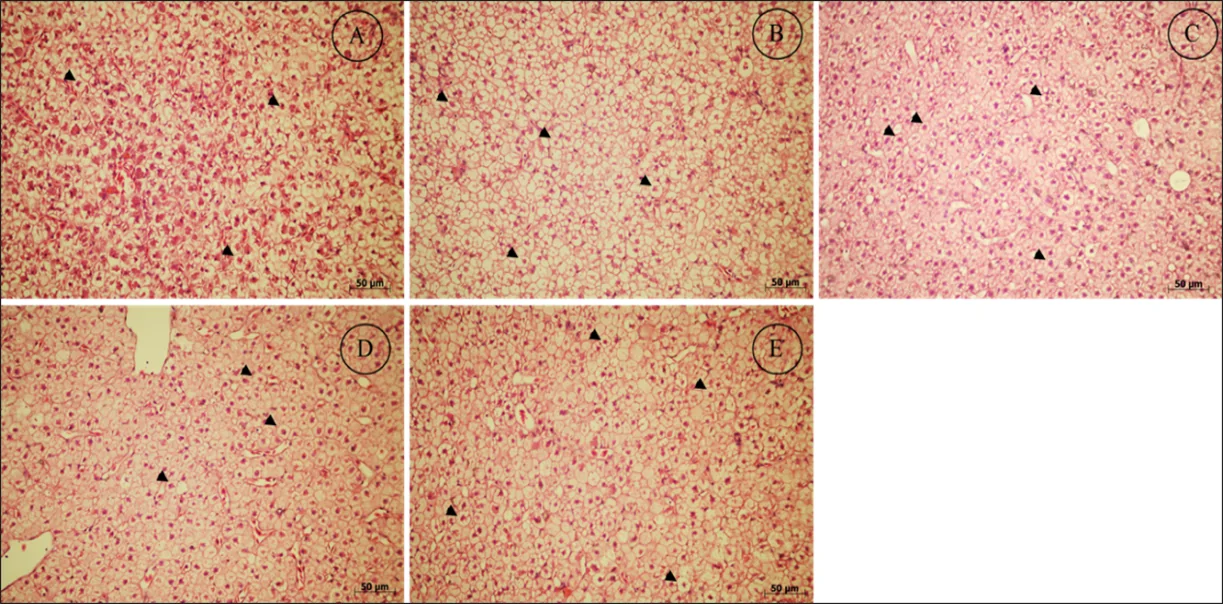
Representative liver histology of goldfish fed diets supplemented with different levels of gac aril powder (GP) and gac aril oil (GO) for 10 weeks (hematoxylin and eosin staining; magnification ×40; scale bar = 50 µm). (A) Control diet without GP or GO supplementation. (B) Diet supplemented with 10 g/kg GP. (C) Diet supplemented with 30 g/kg GP. (D) Diet supplemented with 5 g/kg GO. (E) Diet supplemented with 10 g/kg GO. Black arrowheads indicate hepatocytes with centrally located nuclei. No inflammatory infiltration, hepatocellular degeneration, or vacuolar enlargement was observed in any treatment group.

The liver of fish fed the control diet showed normal architecture, with polyhedral mononucleated hepatocytes containing centrally located nuclei and heterochromatin distributed centrally and peripherally (Figure 2A). A semi-quantitative score of 0, indicating normal liver histology, was assigned to the control group. Similarly, the GP and GO groups showed no significant histological differences compared with the control group. No inflammatory, degenerative, or enlarged vacuolar changes were observed in the examined liver sections (Figures 2B–D). The GP and GO groups also showed a semi-quantitative score of 0, indicating normal liver histology.

## DISCUSSION

### Principal findings

The present study demonstrates the efficacy of GP and, particularly, GO as functional feed additives in fish. Unlike previous investigations in terrestrial animals, this study provides the first evidence in an aquatic species that dietary GO supplementation at 10 g/kg can simultaneously improve pigmentation, carotenoid bioavailability, hematological profile, lipid metabolism, and expression of key immunity-related genes in goldfish. Natural herbs and their extracts have shown substantial benefits in fish nutrition by acting as antioxidants, promoting growth, stimulating appetite, and functioning as immunostimulants [2, 23]. Although several studies have reported the diverse biological functions of gac fruit, including antioxidant, anticancer, anti-inflammatory, antimicrobial, and immunomodulatory properties [23, 37], the specific effects of dietary supplementation with gac aril in fish remain poorly understood. In this study, we evaluated the dietary application of gac aril in powder and oil forms. The results showed that supplementation with GP and GO improved PPV, coloration, total carotenoid deposition, and the expression of immunity-related genes in goldfish. These effects may be attributed to the combined action of the diverse bioactive compounds present in gac arils.

### Growth performance and feed utilization

Growth improvement is an important trait in aquaculture because it is closely associated with productivity and profitability [38]. Several studies have reported positive effects of dietary plants or plant extracts on growth parameters in aquatic species [39, 40]. In the present study, increasing dietary levels of GP and GO did not significantly affect final body weight, weight gain, SGR, FCR, PER, somatic indices, or whole-body composition. However, no adverse effects were observed on growth performance, feed utilization, somatic indices, or whole-body composition. Regarding growth and feed utilization, the findings indicated that 30 g/kg GP was associated with the highest growth response and lowest FCR. In addition, dietary supplementation with GP and GO significantly increased PPV compared with the control group. Although data on dietary gac aril supplementation in fish are limited, the improvement in PPV may be attributed to the enhanced nutritional contribution of GP and GO. Similarly, dietary GP supplementation at 0.2–10 g/kg in chickens had no significant effect on growth or feed utilization [26, 41]. Several carotenoid-rich additives, including turmeric at 2 g/kg, red pepper at 2–3 g/kg, and hericium extract at 1 g/kg, have been reported to improve pigmentation and health status in ornamental goldfish [23, 37, 42].

### Pigmentation and carotenoid deposition

Natural pigmentation is critical in ornamental fish production because it strongly influences consumer preference and market value [21, 22]. Because fish cannot synthesize carotenoids *de novo*, they must obtain these pigments from dietary sources to maintain natural skin coloration, which is an essential quality attribute in high-value ornamental species [21, 22]. In the present study, goldfish fed GP- or GO-supplemented diets showed improved coloration, particularly higher L* and a* values in the head, abdominal, and tail regions compared with the control group. Furthermore, dietary GP and GO increased total carotenoid content in several tissues and increased serum carotenoid levels. Both GP and GO are rich sources of β-carotene, lycopene, lutein, and zeaxanthin (Table 8), although carotenoid contents were generally higher in GO than in GP (Table 8). Goldfish require more than 13 carotenoids, including β-cryptoxanthin, lutein, zeaxanthin, diatoxanthin, alloxanthin, and astaxanthin, for coloration [43, 44]. Therefore, the carotenoids present in GP and GO likely contributed to the enhanced coloration (Tables 5 and 6) and higher total carotenoid levels compared with the control group. To date, only one report has evaluated gac aril for coloration in terrestrial animals, showing that dietary supplementation with 10 g/kg GP enhanced egg yolk color in laying hens [26]. The present study introduces GO as a novel and highly effective natural pigmenting agent for ornamental fish, a finding not previously reported.

**Table 8:** Phytochemical content of gac aril powder and gac aril oil.

**Items (mg/kg)**	**GP**	**GO**
β-carotene	98.34	194.64
Lycopene	65.67	401.16
Lutein	21.37	66.32
Zeaxanthin	3.44	8.16
Astaxanthin	N.D.	N.D.
Flavonoids	281.8	702.1

GP = Gac aril powder; GO = Gac aril oil; N.D. = Not detected.

### Hematological responses

Medicinal plants can positively influence fish hematological indices, although the magnitude of response varies among plant sources and dietary inclusion levels. Hematological indices are important indicators for evaluating health status, stress response, and disease condition in fish [45]. Previous studies have demonstrated a direct association between RBC and WBC counts and immune response in fish, and elevations in these indices are frequently observed after dietary administration of natural immunostimulants [45, 46]. Gac aril contains several active compounds with antioxidant, antibacterial, and immunomodulatory properties [47, 48], which may partly explain the increased WBC count observed in the present study. RBCs act as carriers of Hb, which facilitates oxygen transport, and tissue oxygen delivery depends on RBC maturity and Hb concentration. In this study, dietary supplementation with GP and GO increased RBC, WBC, and Hb levels in a dose-dependent manner in goldfish (p < 0.05). A similar tendency was observed for Hct, although the difference was not significant (p > 0.05), consistent with other studies evaluating medicinal plant supplementation in fish [49, 50]. The improvement in hematological parameters may be related to increased availability of vitamin E, carotenoids, calcium, and iron in the circulatory system [47, 51]. Humans fed GP showed higher Hb content than the control group [14]. Similarly, the high vitamin A content of gac aril has beneficial effects on iron status in rats. Increasing GP levels, accompanied by higher vitamin A intake, may positively affect erythropoiesis and iron mobilization in other vertebrates [52]. However, these mechanisms require further investigation in fish.

### Serum biochemical responses and lipid metabolism

Blood biochemical profiles provide useful information when medicinal plants are incorporated into fish diets. These profiles help assess animal health and monitor metabolic disturbances and diseases caused by biotic and abiotic factors [46]. Albumin, globulin, and the albumin:globulin ratio are components of total serum protein and play important biological roles in fish. In the present study, dietary supplementation with GP and GO did not affect these parameters, indicating that normal biological function was maintained in goldfish. Consistent with these findings, several plant powders or extracts, including *Coriandrum sativum* [53], *Stachys lavandulifolia* Vahl [54], *Rosa canina*, and *Carthamus tinctorius* [55], did not alter serum protein parameters.

Serum AST, ALT, and ALP activities are commonly used as biomarkers in fish because they can indicate disease status and liver injury. In this study, goldfish fed GP and GO diets showed significantly reduced AST levels, whereas ALT and ALP levels did not differ significantly among groups. This result suggests that GP and GO supplementation may have a favorable effect on the health status of goldfish. Previous studies in other vertebrates have shown that gac arils may help alleviate liver disorders [56]. Furthermore, dietary GP and GO supplementation significantly reduced total cholesterol and triglyceride levels, with the lowest values observed in the GO10 group, and increased HDL-c levels in GO-supplemented fish. A previous study demonstrated that dietary gac aril significantly decreased plasma cholesterol, LDL-c, and triglyceride contents in rats [57]. Lin *et al.* [58] reported that mice fed lyophilized gac aril for 10 weeks showed reduced hepatic triglyceride and cholesterol levels. These findings suggest that GP and GO may exert lipid-lowering effects and indicate potential hypolipidemic activity in goldfish.

### Immunity-related gene expression

Inflammatory responses triggered by dietary stimuli can affect tissues and cells. These responses are regulated by cytokines, including pro-inflammatory cytokines, such as *TNF-α*, *IL-1*β, *IL-6*, and *IL-8*, and anti-inflammatory cytokines, such as *IL-10* and *TGF-β* [59, 60]. The pro-inflammatory response is essential for pathogen control, with cytokines regulating innate immunity [60]. As multifunctional regulatory cytokines, *IL-10* and *TGF-*β have anti-inflammatory properties that suppress the production of various cytokines and reduce the adverse effects of pro-inflammatory responses, such as excessive reactive oxygen or nitric oxide production during phagocytosis [61]. Therefore, many studies have suggested that evaluating cytokine expression levels may help predict changes in immune responses [60, 61].

In this study, *TNF-α* and *IL-1*β expression levels were significantly higher in the GP30 and GO groups than in the control group. These findings suggest that GP and GO intake may activate immune responses and improve the ability of fish to respond to pathogen invasion [62, 63]. This effect is likely associated with bioactive compounds present in gac arils, including phenolics, flavonoids, and carotenoids, which may promote the expression of *TNF-α*, *IL-1*β, and *IL-10* mRNA. Previous studies have shown that these bioactive compounds can upregulate the expression of immune-related genes [64–66]. In addition, carotenoids serve as precursors of vitamin A, and carotenoid deficiency negatively affects antioxidant responses and other protective health mechanisms in fish [67]. Dietary supplementation with flavonoids or carotenoids has also been shown to enhance antioxidant and anti-inflammatory capacity in several fish species [68–70].

### Lysozyme and innate immunity

Granulocyte-secreted lysozyme is an important immune component that enhances innate immunity by degrading bacterial peptidoglycans and activating the complement system, phagocytic cells, and other immune responses [71]. In the present study, lysozyme mRNA expression was upregulated in fish fed GP- and GO-supplemented diets compared with the control group. This upregulation suggests a positive effect on innate immune responses. Tahir *et al.* [72] reported that dietary lycopene supplementation significantly increased lysozyme activity in common carp (*Cyprinus carpio*) compared with the control group. Similarly, Mousavi *et al.* [73] reported increased lysozyme activity in red fantail male guppy (*Poecilia reticulata*) after supplementation with carotenoid-rich herbs. In addition, dietary enrichment with β-carotene improved non-specific immune responses in goldfish, as demonstrated by increased total immunoglobulin and lysozyme activity [74]. Recent studies have also shown that flavonoid-rich plants can significantly enhance lysozyme activity and mRNA expression levels in various aquatic species [75–77]. Based on these findings, dietary supplementation with GP and GO may stimulate immune- and cytokine-related gene expression, thereby improving the health status of goldfish. However, pathogen challenge trials are required to confirm the protective efficacy of GO supplementation and substantiate its immunostimulatory effects in ornamental fish. The differential modulation of pro- and anti-inflammatory cytokines and lysozyme gene expression by GP and GO provides new information regarding the immunomodulatory potential of gac-derived products in fish, extending beyond the antioxidant and anti-inflammatory effects previously documented in mammals.

### *HSP-70* expression and stress response

*HSP-70* is a stress-responsive gene associated with stressors such as heat, oxidative stress, starvation, high stocking density, and pollutant exposure [78]. Therefore, under normal rearing conditions, dietary gac supplementation may not markedly affect *HSP-70* expression. Gac is rich in antioxidant compounds, including carotenoids, lycopene, and flavonoids, which help maintain cellular homeostasis and reduce oxidative stress. Consequently, the *HSP-70*-related stress response pathway may not have been activated in the present study. Similar findings have been reported in fish maintained under non-stress conditions or with adequate antioxidant protection [79]. Previous studies have shown that *HSP-70* expression increases under stress conditions such as temperature fluctuation, handling, heavy metal exposure, starvation, and high stocking density [78].

### Liver histology and safety evaluation

The liver is the largest internal organ in fish and plays a central role in metabolism. It is also a major target organ affected by biological and environmental factors, including feed, pollutants, toxins, parasites, and microorganisms [80]. The microstructural morphology of hepatocytes can directly reflect nutrient absorption and abnormal growth and is therefore useful for assessing fish nutritional status [81]. In this study, dietary supplementation with GP and GO did not alter liver histology (Figures 2A–D). This finding was further supported by unchanged HSI and favorable AST and ALT responses, suggesting that GP and GO did not exert harmful effects on hepatocytes. Studies in terrestrial species have shown that gac aril contains higher lycopene levels than many other fruits and contains antioxidant compounds that may protect tissues, including the liver, colon, and breast, during cancer development [82, 83]. Importantly, this study is the first to confirm the safety of gac aril products on liver morphology in fish, supporting their potential as safe and sustainable feed additives.

## CONCLUSION

Dietary supplementation with GP and GO was safe and well tolerated by goldfish throughout the 10-week feeding trial, with no adverse effects on survival, growth performance, somatic indices, whole-body composition, or liver histology. Although neither GP nor GO significantly enhanced growth, supplementation improved PPV, indicating more efficient protein utilization. More importantly, both GP and GO markedly enhanced body coloration and carotenoid deposition, with GO generally producing greater improvements in carotenoid accumulation in the muscle, liver, and serum than GP. Dietary GO also improved hematological parameters by increasing RBC, WBC, and Hb levels, favorably modulated lipid metabolism through reductions in AST, total cholesterol, and triglycerides together with increased HDL-c, and significantly upregulated the expression of key immunity-related genes, including *TNF-α*, *IL-1β*, *IL-10*, and lysozyme, without altering *HSP-70* expression. These findings indicate that GO has greater biological efficacy than GP, likely due to improved bioavailability of lipid-soluble carotenoids and other bioactive compounds.

From a practical perspective, GO represents a promising natural feed additive that could serve as a sustainable alternative to synthetic carotenoid pigments and potentially reduce dependence on chemotherapeutic agents in ornamental aquaculture. The simultaneous improvement in pigmentation, carotenoid bioavailability, hematological status, lipid metabolism, and immune-related gene expression highlights its potential to enhance both the aesthetic quality and overall health of ornamental fish, thereby increasing their commercial value.

A major strength of this study is that it provides the first comprehensive comparison between powder and oil forms of gac arils in an ornamental fish species, integrating evaluations of growth performance, pigmentation, carotenoid deposition, hematological and biochemical responses, liver histology, and immunity-related gene expression within a single experimental framework. This holistic approach provides novel evidence supporting the use of gac-derived products as multifunctional feed additives in aquaculture.

Nevertheless, the study has several limitations. Protective efficacy against infectious diseases was not evaluated through pathogen challenge experiments, antioxidant enzyme activities and oxidative stress biomarkers were not determined, and the molecular mechanisms underlying carotenoid absorption, transport, and metabolism were not investigated. Furthermore, the trial was conducted in a single ornamental fish species under controlled laboratory conditions.

Future studies should evaluate the long-term effects of GP and GO supplementation across ornamental and food fish species, assess their effects under commercial production conditions, investigate the molecular pathways regulating carotenoid metabolism and immune modulation, and validate their protective efficacy through pathogen challenge trials. Additional studies should also assess the economic feasibility of incorporating GO into commercial ornamental fish diets.

Overall, the present study demonstrates that dietary GO, particularly at 10 g/kg, is a highly effective natural functional feed additive that enhances pigmentation, carotenoid deposition, hematological status, lipid metabolism, and immune responses while maintaining normal liver morphology and overall fish health. These findings support the potential of GO as a safe, sustainable, and value-added nutritional strategy to improve the health and market quality of ornamental fish.

## DATA AVAILABILITY

The datasets generated and/or analyzed during the current study are available from the corresponding author upon reasonable request.

## AUTHORS’ CONTRIBUTIONS

AK: Conceptualization, methodology, project administration, supervision, formal analysis, investigation, writing of the original draft, and review and editing of the manuscript. WN and NA: Formal analysis and investigation. NM, KR, WI, KW, and PT: Methodology, conceptualization, and manuscript review. All authors have read and approved the final version of the manuscript.
